# Supplementary material for: Healthy eating and physical activity: Analysing Soweto’s young adults’ perspectives with an intersectionality lens
Source: PLOS Glob Public Health. 2023 Jul 19;3(7):e0001429. doi: 10.1371/journal.pgph.0001429 (PMC10355453; doi:10.1371/journal.pgph.0001429)
Supplement: S1 File — (DOCX) [file pgph.0001429.s001.docx]

**S1 File**

Focus Group Discussion Guide: Citizen Scientist’s barriers and solutions to healthy eating and physical activity

1. How do you define health? What makes you healthy?
2. How do you define healthy living? What makes it difficult to be healthy?
3. What do you understand about nutrition and physical activity?
4. What key areas do you think should be prioritised to address challenges of or improve nutrition? What solutions or interventions do you suggest to solve these challenges?
5. How is the way we eat related to our health? How important is it? Can you have a bad diet and still be healthy?
6. What key areas do you think should be prioritised to address challenges of insufficient physical activity? What solutions or interventions do you suggest to solve these challenges?
7. How do you think physical activity is related to health? How important is it? Can you be physically inactive and still be healthy?
8. What do you understand by obesity? Is it a problem in your community?
9. What are the causes of obesity? What are the risks associated with obesity?
10. Is there any relationship between nutrition, physical activity and obesity?
11. What can be done to address the prevalence of obesity and improve health in your community?
12. Are there any programs available that you know that focus on improving health in your community? Give some examples and what they do.
13. Who do you think should be involved in finding solutions to address challenges of nutrition, physical activity and obesity in your community? Why?
