# Supplementary material for: Healthy eating and physical activity: Analysing Soweto’s young adults’ perspectives with an intersectionality lens
Source: PLOS Glob Public Health. 2023 Jul 19;3(7):e0001429. doi: 10.1371/journal.pgph.0001429 (PMC10355453; doi:10.1371/journal.pgph.0001429)
Supplement: Attachment — Submitted filename: PGPH-D-22-01947 - Response to reviewers.docx [file pgph.0001429.s003.docx]

PGPH-D-22-01947 – Response to reviewers comments
Healthy eating and physical activity: analysing Soweto's young adults' perspectives with an intersectionality lens
PLOS Global Public Health

| Reviewers comments to authors | Authors response |
| --- | --- |
| Reviewer 1 |  |
| The Authors should provide more detail on the following:  Table 1- the authors should explicitly how the descriptive statistics are displayed: mean and standard deviation, and n and percentages. This could be in a footnote. | Thank you for this important comment. A statement stating how descriptive statistics are presented in Table 1 is now added. |
| Methods  • Page 5: Study Setting and Design: o The setting (Lines 95-104) is well-defined but the design, from line 106, the paper should speak to the paradigm e.g. interpretivism, and also indicate what theory, theory, framework or literature informed the interview guide | From lines 140 to 174, the theoretical framework that guided this study is explained. |
| o The “Participants and recruitment” section should be expanded to include the inclusion/exclusion criteria and whether other factors besides household income were used to achieve maximum variation within the sample e.g., ethnicity, religion | Thank you; the participant recruitment section has been updated with more information on the inclusion/exclusion criteria. |
| • Page 8: “Theoretical framework” o Line 162: the author should indicate if Figure 1 is original or of it is adapted or adopted from another source | The manuscript now includes a statement stating that Figure 1 is original. |
| “Data collection”: Line 168: please indicate the online modality/platform used for the remote interviewing | There is now more information on the online platform used for remote interviews. |
| o Line 170: Give the value of the voucher in an international currency e.g., USD$ and indicate how this compared to average weekly income for example to enable the reader to see the extent to which this was an appropriate incentive | The dollar value of the airtime recharge voucher is now provided. Ethically, we are not permitted to offer incentives to participants; therefore, the amount provided was intended solely to facilitate participation in the online discussion and was more than sufficient for its duration. |
| Line 174: Please give the rationale for the gender separation to enhance the reader's understanding of the context/study design | To clarify the justification for gender separation during the discussions, the phrase "in order to maximise disclosure among focus group participants" has been added. |
| Lines 177- 178: Please clarify the apparent conflict between the statement “Audio recordings were transcribed verbatim and, when necessary, translated into English” and the statement in Line 166; "The FGDs were conducted in English…” | Our participants are not native English speakers, so we permitted them to use their native language when they were unable to express themselves clearly in English. |
| Reviewer 2 |  |
| Line 33. You could add in how many men and how many women in addition to the total sample size. | The number of men (n= 15) and women (n= 15) who participated is now added to the total sample size (n= 30). |
| Line 35. It would be helpful to have something more specific regarding ‘such behaviours’ e.g, ‘barriers to engaging healthy diet and physical activity behaviours’ | Now added are specific examples of barriers to healthy eating and physical activity that were previously mentioned. |
| Line 38. I think these results could be expanded a little here/ developed further in relation to interpretation. What does it mean when young men are describing themselves as lazy? | More information is now provided to explain why the young men described themselves as lazy. |
| Results: Are these examples of barriers or the only barriers that were described? | The barriers cited in this paper are those outlined by participants. Table 2 of supplementary file 2 can be useful for providing additional clarity. |
| Introduction: - Some explanation of how urbanisation, globalisation and commercial health determinants are indirectly affecting health, particularly in LMICs would be helpful to include here. | Thank you for your comment. However, lines 50 to 54 of the introduction explain how urbanization, globalization, and commercial determinants of health are associated with the rise of non-communicable diseases in LMICs such as Africa. |
| Sentence on line 54-55 needs rephrasing to improve clarity. | To clarify the sentence, more information has been added. |
| Could you add a line at the end is needed for why you have chosen to use an intersectional lens to explore these barriers? I know you discuss this more in the methods so something brief is fine. | A line emphasizing the significance of adopting an intersectionality perspective in this study has been added per your suggestion. |
| Methods: - Line 106: You could state the study design somewhere at the start of the method. | The study design is now added at the start of the methods section. |
| - It is not at all clear what was the purpose of collecting anthropometric data (e.g., weighing participants)- rationale for this isn’t provided but it is important. What was the use of these measures being taken for a qualitative study? If you needed this information, could you not have asked them for their BMI? There also isn’t any ethical considerations about this- how did you ensure that participants were comfortable with being weighed, for example. | The significance of the anthropometric measurements is clearly highlighted at the start of the results section, which is the participants' characteristics. We were unable to simply ask participants about their BMI status, and all participants provided consent during recruitment to prove that they were keen to be measured. |
| Participants: Did you purposively sample? You state that you recruited from both low and high-income households- so did you aim for homogenous or heterogenous FGDs in relation to this? | Yes, we purposefully selected a sample of participants for this study. This was emphasized in the methods section. The aim was to conduct FGDs with diverse gender and socioeconomic representation. |
| 135: ‘how health-related behaviours turn out’ needs to be rephrased to improve clarity. | The sentence is now rephrased for better clarity. |
| - 138-39: This sentence needs rewording for clarity. In addition, be sure to use qualitative terms like ‘explore’ rather than assess which is more quantitative. | The word 'assessed' has been replaced with the word 'explored' in this sentence. |
| Line 167- a comma is needed after qualitative researchers | Thank you for the suggestion, a comma is now added. |
| What questions did you ask in the FGDs? | Supplementary file 3 Appendix 1 is our interview guide which contains a list of questions we asked during the FGDs. |
| Results: - Overall, the result section is very long. I think quite a bit of this can be focussed down and collapsed to reduce this section. | We acknowledge that the results section is somewhat lengthy; however, all of the reported information is essential for elucidating the importance of applying an intersectionality perspective in this paper. |
| Numbers of men and women needed at the beginning (or a table could provide participant details perhaps) | The number of men and women who participated in the study has been added to the start of the results section. A supplementary file 1 Table 1 detailing the characteristics of each participant is also included. |
| I can’t see anywhere where you use the anthropometric data. So what was the purpose of collecting it? | The importance of the anthropometric data is now reflected at the beginning of the results section. |
| Domain 1- it seems to be as though your participants had a good understanding rather than basic of health in relation to sleep, check-ups, diet etc to maintain wellbeing. | The word ‘basic’ is now replaced with ‘good’. |
| Line 263- this sub theme ‘inability to apply nutrition knowledge’ is a little bit confusing. On one hand you say that your participants have the knowledge they need regarding nutrition. However, you then go on to suggest nutrition education. It seems as though they know what to do, but there are other barriers that are getting in the way. | Yes, participants stated that while they understand that fruits and vegetables are good for their bodies, they do not necessarily understand what a balanced diet is or what nutrients they get from specific foods, highlighting the need for nutrition education so that they can translate it into healthy behaviour action. The solutions are also proposed by participants. |
| Some of the theme names could be developed a bit further e.g., ‘priority is for everyone to eat’. An example would be developing this a bit further to incorporate something about financial constraints resulting in priority being to feed all family members as cheaply as possible. | This theme is expanded to read as 'Only mom works, priority is for everyone to eat' to emphasize the financial constraints and the fact that only one person bears the majority of the burden of providing food for the family. |
| The theme ‘eating healthy means sickness’ is very interesting! Were there any other social influences outlined? Individuals in this age group are often heavily influenced by peers/ social norms. | Yes, thank you for the positive feedback. In our discussions, the perceptions of peers and community members regarding healthy eating emerged as the primary factor influencing the food choices of the majority of young people. |
| Discussion: - The diagram is really nice on page 19. I think this could potentially go at the start of your findings section where you first introduce your themes? | We believe the placement of the diagram in the discussion section is appropriate because it helps the reader understand the intersectionality of the various barriers influencing young adults' choice of behaviour. |
| Motivation (or lack of) was a barrier suggested by your participants. There is a lot of literature on motivation to engage in health behaviours so this could be referenced in your discussion section perhaps to address this individual factor. | Thank you for the comment, however, the primary objective of this paper was not to emphasize individual barriers, but rather to emphasize the interconnectedness of the various factors and the need to consider them collectively. |
|  |  |
